# Native Mitral Valve Infective Endocarditis From Flossing: A Case Report and Emergency Department Management

**DOI:** 10.7759/cureus.12144

**Published:** 2020-12-18

**Authors:** Rachel E Bridwell, Neil P Larson, Sara Birdsong, Brit Long, Sarah Goss

**Affiliations:** 1 Emergency Medicine, Brooke Army Medical Center, Fort Sam Houston, USA

**Keywords:** infective endocarditis, native mitral valve, flossing, streptococcus gordonii

## Abstract

Infective endocarditis (IE) is a rare, elusive disease, carrying a 10%-30% mortality. Requiring a high index of suspicion, IE affects damaged native valves and prosthetic valves. While there are a number of inherent risk factors that predispose patients to IE, dental work in the preceding six weeks is often a culprit of disease, colonizing damaged native mitral valves with *Streptococcus viridans* species. Traditionally, flossing has been suggested to be protective against IE. We present a case of *S. gordonii* subacute IE on a regurgitant native mitral valve secondary to vigorous flossing.

## Introduction

Native mitral valve infective endocarditis (IE) represents a small but important subset within IE. IE is often classified into left (mitral and aortic) and right (tricuspid and pulmonic) sided etiologies as well as by the nature of the valve affected (native or prosthetic) [1]. IE holds an incidence of three-nine cases per 100,000 persons, with 40% of these cases affecting the mitral valve [2-3]. Despite its low incidence, IE is crucial to diagnose early, due to a 10%-30% mortality [4]. While the mitral valve does not experience the same high pressures and subsequent rates of calcification as the aortic valve, chronic regurgitation and prolapse of the two leaflet structure wears the valve and predisposes it to vegetation growth [5]. A variety of risk factors for bacteremia can increase the likelihood of native mitral valve IE, however, there is no current literature to implicate flossing as a risk factor. We present a case of flossing-induced mitral IE on a native valve.

## Case presentation

**Figure 1:**
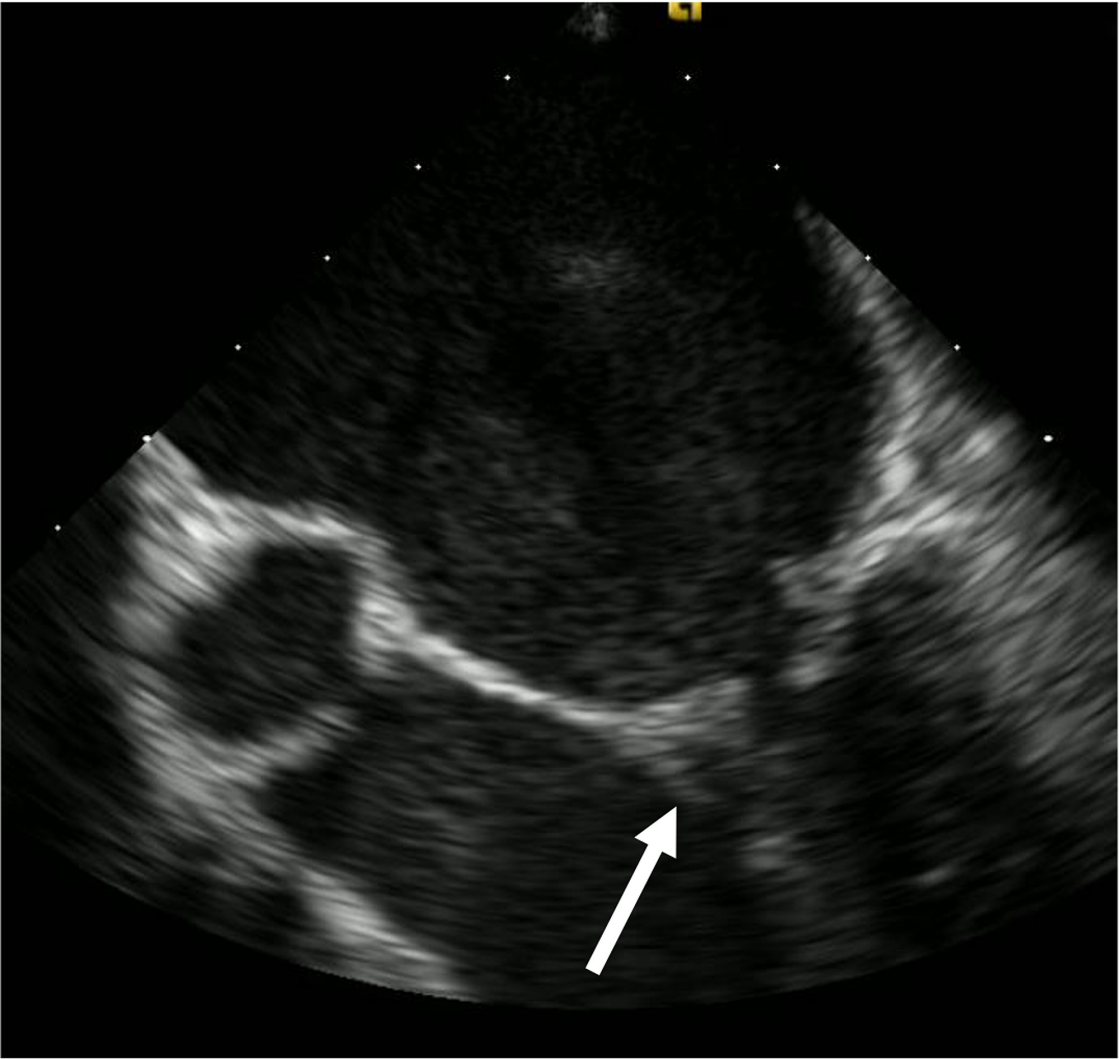
Transesophageal echocardiogram demonstrating a small independently mobile linear echo-density on the posterior leaflet of the mitral valve (white arrow), consistent with a vegetation.

A 63-year-old male with a pertinent past medical history of mitral regurgitation presented to the emergency department (ED) for three weeks of fevers, measured at 103.0 degrees Fahrenheit nightly. He was previously evaluated in an outside ED and by his primary care manager during this time, but his fevers persisted without an identified source. The patient denied recent dental work or colonoscopy, IV drug use, alcohol use, hemodialysis, or previous valvular replacement. However, the patient endorsed vigorous flossing with bloody gingivae. Review of systems was otherwise unremarkable. The patient’s initial vitals were within normal limits. Exam did not reveal a murmur, skin lesions, or nodules. Electrocardiogram and chest radiograph did not demonstrate any abnormalities. Laboratory evaluation revealed a white blood cell count of 10,100 cells/uL, with an elevated C-reactive protein of 14.00 mg/dL and erythrocyte sedimentation rate of 95 mm/h. Complete metabolic panel, urinalysis, and thyroid stimulating hormone were within normal limits. The patient was empirically started on 1.5 g of vancomycin and 3.375 g of piperacillin/tazobactam intravenously. Three separate blood cultures collected 15 min apart yielded Gram-positive cocci, which speciated to *Streptococcus gordonii*. Following an unremarkable transthoracic echocardiogram (TTE), a transesophageal echocardiogram (TEE) revealed a small vegetation on the posterior leaflet of the mitral valve (Figure 1). Based on sensitivities of the *S. gordonii*, antibiotics were narrowed to 242 mg of IV gentamicin every 24 h and 24 million units of penicillin G continuous infusion. On hospital day three, the patient’s blood cultures no longer grew *S. gordonii*, and he was discharged home on two weeks of outpatient gentamicin infusions and four weeks of penicillin infusions.

## Discussion

Infective endocarditis is a rare but critical diagnosis that is challenging to identify due to its various subtle presentations and underlying risk factors. These risk factors traditionally include IV drug use, valvular heart disease, implantable cardiac devices, indwelling lines, unrepaired cardiac abnormalities, recent dental work, or immunocompromised state (e.g., hemodialysis, chronic renal/liver disease, etc.) [6]. Damaged valves generate nonlaminar flow which predisposes them to platelet aggregation and subsequent bacterial colonization [7]. While S. aureus is the most common cause of IE overall, native mitral endocarditis secondary to dental etiology is still most commonly caused by *S. viridans* species [8].

In evaluating patients for IE in the ED, current fever or history of recent fever is the most common presenting symptom, occurring in up to 80% of cases [9]. Dental procedures in the six weeks preceding ED presentation increase the risk of native mitral valve IE [10]. While toothbrushing in a patient with poor dental hygiene has been estimated to increase bacteremia risks far more than a single tooth extraction, there are no data to suggest the same incurred risk for flossing [11]. In fact, flossing has been associated with a decreased risk of IE, highlighting the unique nature of this case [12].

On exam, eliciting either a murmur or history of a murmur is also helpful as this feature is a known marker of valvular damage and present in 10% of cases [13]. In subacute native mitral valve IE, classic physical exam findings of cutaneous and ocular embolic phenomenon (e.g. Osler nodes, Janeway lesions, etc.) are rare, occurring in only 5%-8% of cases [14]. However, in left sided lesions, 20%-30% of patients may present with stroke like symptoms or cerebrovascular accidents [14].

The cornerstone of laboratory evaluation of IE is blood cultures, requiring three separate peripheral cultures drawn at different times within one hour, though these will yield limited utility in the ED. However, blood cultures with an identified organism growing within 14 h portend increased mortality [15]. An electrocardiogram should also be performed in patients with concern for IE to assess for new atrioventricular blocks or conduction abnormalities.

Empiric treatment of native valve mitral endocarditis should ideally target any risk factors, starting with broad antibiotic coverage with vancomycin and ceftriaxone; in those with native mitral valve involvement, suspected dental etiology, and normal renal function, penicillin G 12-24 million units intravenously per day or ceftriaxone 2 g intravenously per day in combination with gentamicin 3 mg/kg intravenously per day is the American Heart Association recommended antibiotic coverage [16]. Embolic phenomena are more common with aortic and mitral valve lesions greater than 1 cm, especially during the first two weeks after initiating IV antibiotics [17-18].

Patients should be admitted to the hospital for further antibiotic administration as well as comprehensive TTE and TEE, as TTE alone is insufficient for assessment of IE [9]. Performed in conjunction within 12 h of admission, these sonographic modalities will identify 90% of all vegetations [19]. TEE is especially useful when assessing the posterior leaflet of the mitral valve and the left ventricular outflow tract [9].

The above case represents an unusual etiology of a rare disease. One previous case report demonstrated similar presentation and antimicrobial management in *S. gordonii* IE, though this case was secondary to incision and drainage of a dental abscess [20]. While *S. gordonii* is a prominent member of the *S. viridans* group, there are no cases to date of IE secondary to flossing.

## Conclusions

Though less commonly affecting native valves, IE represents a challenging but dangerous clinical entity that requires a high index of suspicion. High risk features include IV drug use, recent dental scaling or care, prosthetic valves, or native valves incurring damage. However, no previous case has been described with flossing-induced IE on a native mitral valve. Emergency clinicians should consider this diagnosis in patients with high risk features as well as recurrent fevers without an identified source.
